# Epidemiology of Hepatitis E Virus Infection in Patients on Chronic Hemodialysis

**DOI:** 10.5812/jjm.6993

**Published:** 2014-05-01

**Authors:** Seyed Seifollah Beladi Mousavi, Farzad Motemednia, Marzieh Beladi Mousavi

**Affiliations:** 1 Chronic Renal Failure Research Center, Department of Internal Medicine, School of Medicine, Ahvaz Jundishapur University of Medical Sciences, Ahvaz, IR Iran; 2 Department of Chemistry, Islamic Azad University of Omidiyeh Branch, Omidiyeh, IR Iran

**Keywords:** Hepatitis E, Kidney Failure, Chronic, Renal Dialysis, Iran

## Abstract

**Background:**

Many studies have been done on the epidemiology of Hepatitis E on general population, but the data among patients with end stage renal disease (ESRD) are few and give conflicting results.

**Objectives:**

The aim of this study was to investigate the prevalence of hepatitis E virus (HEV) infection and its relationship in ESRD patients undergoing maintenance hemodialysis (HD).

**Patients and Methods:**

This cross-sectional study was carried out on ESRD patients treated with HD in Imam Khomeini Hospital, Ahvaz city, Southwest of Iran. Blood sampling of patients was collected immediately before the dialysis session and the serum were evaluated for anti-HEV IgG titers by enzyme-linked immunosorbent assays. The statistical package for social sciences (SPSS) version 15 software was used for data analysis.

**Results:**

Out of 47 ESRD patients, 27 were male (57.4%) and 20 were female (42.6%), with mean age of 55.27 ± 8.1 years. The prevalence of anti-HEV antibody was 10.6 % (five patients, four male and one female). The mean age of HEV positive and negative patients were 58 ± 5.52 and 53.82 ± 15.55 years, respectively without any significant difference (P = 0.058). There also was no significant association between HEV and gender (P = 0.28). The mean time of HD in HEV positive and negative patients were 1224.2 and 1168.5 days, respectively with no significant association (P = 0.88). In addition, there also was no association between HEV and HCV (P = 0.61).

**Conclusions:**

According to the present study, the prevalence of anti-HEV IgG antibody was 10.63 % among chronic HD patients and there was no association between HEV, age, gender, duration of HD and HCV antibody titer.

## 1. Background

Hepatitis E virus (HEV) is a non-enveloped, positive-sense single-stranded RNA virus that is approximately 27 to 34 nm in diameter. It has been classified as the single member of the genus Hepevirus and has a similar structure to the viruses of the *Caliciviridae* and *Tombusviridae* families. This virus was first discovered during an outbreak in New Delhi, India, in 1955 (1-3). Hepatitis E is an important public health concern in regions with low sanitation standards that promote the transmission of the virus. It also may be more prevalent in developed countries. Transmission of HEV primarily occurs by the fecal-oral route through contaminated water or food similar to that of hepatitis A virus. Vertical transmission, blood transfusions, person-to-person contact are not considered as important routes of HEV transmission, however some studies have indicated that they play role in HEV transmission, particularly in endemic areas (4-7).

Although HEV generally causes a self-limited acute infection and mortality rates are generally lower, acute severe liver disease and fulminant hepatitis can occur, resulting in an overall fatality rate of 0.5 to 3 percent and as high as 20% in pregnant women (8-10). There are many studies about epidemiology of HEV in general population, but the data among patients with end stage renal disease (ESRD) are few and give conflicting results (11-15); therefore further studies are needed.

## 2. Objectives

In the present study, we have conducted an epidemiologic study to investigate the prevalence of HEV infection and its relationship among ESRD patients under hemodialysis (HD) in Imam Khomeini Hospital, Ahvaz, Iran.

## 3. Patients and Methods

In a cross-sectional study, ESRD patients treated with hemodialysis living in the province of Khuzestan, Iran enrolled for the study. The ESRD was defined as irreversible and permanent loss of renal function due to any causes requiring hemodialysis (HD). In this study, 2-4-hour HD sessions performed two or three times a week, using semi-synthetic (cellulose diacetate), or synthetic (polysulfone) dialyzer membranes, with blood flow rate of 200-400 mL/min and the dialysate flow rate of 500 mL/min and bicarbonate-based dialysis solution at a delivered bicarbonate concentration of 35-40 mEq/L. A standardized questionnaire was used to collect social and demographic data, causes of ESRD, date of onset of starting HD and laboratory data.

### 3.1. Laboratory Assay

Blood samples of patients were collected immediately before the dialysis session and the serum was separated without delay. Sera were coded and evaluated for anti-HEV IgG by enzyme-linked immunosorbent assays (Diapro, Italy) according to the manufacturer's instruction. To confirm the initial results of the study, all positive samples with anti-HEV IgG were retested in duplicate with the same EIA assay. In addition, all participants were previously screened foranti-HCV (Third generation assay, Diasorin, USA), HBs Ag (Abbott Laboratories, and North Chicago, IL, USA) and anti-HIV (Biotest, Germany) by EIA assay.

### 3.2. Statistical Analysis

At the end of the study, the statistical package for social sciences (SPSS) version 15 software was used for analysis. The prevalence rates and 95% confidence intervals were calculated. Chi-square tests, Fisher’s exact and T-test were performed to test the association of quantitative and qualitative variables in the anti-HEV IgG positive and negative patients. Statistical significance was considered at the P value of < 0.05 in all statistical analyses.

## 4. Results

Overall, 47 ESRD patients, 27 male (57.4%) and 20 female (42.6%), with mean age of 55.27 ± 8.1 years, undergoing maintenance HD in the hemodialysis center of Imam Hospital, Ahvaz city, Southwest of Iran were enrolled for the study. The causes of ESRD were as follow: unknown in 26.4%, hypertension in 24.3%, diabetes in 23.1%, glomerulonephritis in 13.5%, obstructive uropathy in 8.8% and cystic kidney disease in 3.9% of cases. Our HD centers did not administer patients with HbsAg positive. The prevalence of anti-HEV antibody among our ESRD patients was 10.6 % (five patients, four males and one female). The mean age of patients with HEV positive and negative were 58 ± 5.52 and 53.82 ± 15.55 years with no significant difference (P = 0.058). Although 80% of patients with HEV positive and only 54.8% of HEV negative were male, but there was not a statistically significant difference between males and females (P = 0.28).

To evaluate the association between duration of HD and HEV positivity, we determined the duration of HD in both groups. The mean time of HD in patients with HEV positive and negative were 1224.2 and 1168.5 days respectively and there also did not have any significant association (P = 0.88). Of 47 patients two patients (4.3%) had HCV antibody, both were HEV negative and there was not any association between HEV and HCV (P = 0.61). Other characteristics of HEV positive and negative patients are summarized in Table 1.

**Table 1.** Characteristics of Patient With ESRD Underwent Hemodialysis With and Without Anti-HEV IGg Antibody ^a^

Characteristics	Anti-HEV Antibody		P Value
	Positive (n = 5)	Negative (n = 42)	
**Mean age, y**	58 ± 5.52	53.82 ± 15.55	0.058
**Gender**			
Male	4 (80)	23 (54.8)	0.28
Female	1 (20)	19 (45.2)	0.28
**Duration of hemodialysis, d**	1224.2 ± 156	1168.5 ± 247	0.88
**Hemoglobin level, mg/dL**	10.33 ± 1.7	10.5 ± 0.7	0.82
**Serum Creatinine level**	9.34 ± 2.68	9.51 ± 2.6	0.89
**Serum Calcium level, mg/dL**	8.33 ± 0.51	7.94 ± 0.77	0.13
**Serum Uric acid level, mg/dL**	6.360 ± 0.81	22.25 ± 0.76	0.78
**Serum phosphor level, mg/dL**	5.76 ± 0.38	5.70 ± 0.54	0.83

^a^ Data are presented in Mean ± SD or No. (%).

## 5. Discussion

Evaluation of viral hepatitis among patients undergo HD and comparing those in other centers in same and or other countries is a good guide to better resolve the problem of these patients. Although, there are many studies about the epidemiology of HBV and HCV infections among patients with ESRD in both developed and developing countries

but the findings about HEV infection are limited and the results are conflicting. It seems that the prevalence of HEV infection among patients with ESRD is relatively independent from the prevalence of HEV in the general population and it may vary in different hemodialysis units in developed and the developing countries (16-22).

In a study from Japan, Mitsui et al. investigated the prevalence of hepatitis E virus infection among ESRD patients who had been underwent hemodialysis for 7.6±6.3 years. The sera of these patients were tested for detecting the IgG antibodies against HEV by an enzyme-linked immunosorbent assay at the start of hemodialysis. The prevalence of anti-HEV antibody was 9.4% and in the end of the study, the author concluded that the prevalence of de novo HEV infection during hemodialysis is low (16).

The result of Stefanidis et al. (17) demonstrated that the prevalence of the virus is lower than our findings. In addition their study shows that the prevalence of anti-HEV among patients with ESRD who were treated in the five HD units of central Greece was 4.8% varying from 1.8*-*9.8%. The authors found no association between anti-HEV positivity and age, gender, duration of HD, the serologic marker of HBV or HCV and history of transfusion (17).

The prevalence of the virus is mildly higher in the present study compared to the results of Mitsui et al. (16) and Stefanidis et al (17). According to our study, the prevalence of anti-HEV IgG antibody was 10.63 % among chronic HD patients in the HD center of Imam Hospital in Khuzestan province, Southwest of Iran; indicating that HEV infection is endemic in our country. In addition, similar to the result of Stefanidis et al. (17) any association between HEV, age, gender, duration of HD and HCV antibody were observed in our study. There are a few studies about the epidemiology of HEV in HD patients and renal transplant recipients in Iran with different results, for example, Taremi et al. (20) evaluated the prevalence of anti-HEV antibody in three different HD units in Tabriz, Northwest of Iran. In this study, the prevalence of anti-HEV IgG antibody was 7.4 % with no significant association between HEV, age, gender, duration of hemodialysis, history of transfusion and blood borne infections (HBV, HCV and HIV).

In another study, Rostamzadeh Khameneh et al. (21) investigated seroprevalence of HEV among 91 renal transplant patients who were randomly selected in Urmia, the other North-Western region of Iran. In this study significant number of patients who had received a kidney transplant (30.8%) had anti-HEV IgG antibody compared to the previous studies and also our study among HD patients. At the end of study, author concluded that the prevalence of HEV among patients who had received a transplant is independent to the prevalence of HEV in the general population. Although, in a few reports, there is a gender tendency toward females in the prevalence of HEV infection among patients underwent maintenance HD, however in the majority of studies about HEV infection, the seroprevalence of the virus is higher in males compared to females (15, 22, 23). There also was a gender tendency toward males in our study; however it was not statistically significant.

Hepatitis E is an important public health concern in developed and developing countries. Although, transmission of HEV primarily occurs by the fecal-oral route, some studies have indicated that vertical transmission and blood transfusions may involve in the transmission of HEV, particularly in endemic areas. Patients with ESRD underwent maintenance HD may also be at risk of HEV. According to the present study, the prevalence of anti-HEV IgG antibody was 10.63 % among patients with chronic HD and we did not find any association between HEV, age, gender, duration of HD and HCV antibody in the HD Center of Imam Hospital in Khuzestan province, Southwest of Iran.
